# Sonodynamic therapy for treatment of C6 glioma in a rat model step 1: feasibility of tumor model

**DOI:** 10.1186/2050-5736-3-S1-O10

**Published:** 2015-06-30

**Authors:** Paul Schmitt, Jessica Foley, Neal Kassell, Jason Sheehan, Zhiyuan Xu

**Affiliations:** 1 University of Virginia, Charlottesville, VA, United States; 2 Focused Ultrasound Foundation, Charlottesville, VA, United States

## Background/introduction

Gliomas represent the most common and devastating primary CNS tumor. While surgical resection is the mainstay of treatment, treating physicians are seeking a safer and more effective treatment modality that is non-invasive, deep-penetrating, and less prone to infection and damage to surrounding tissues. Photodynamic therapy, utilizing a photosensitizing agent with laser light, while effective in a variety of solid tissue tumors, has not proven to be especially advantageous in the treatment of gliomas, due to the poor penetration of the light. Sonodynamic therapy has been explored as an alternative to PDT. Sensitizers such as 5-aminolevulinic acid (5-ALA) and indocyanine green (IcG) have been shown to be preferentially taken up by glioma cells. Low-intensity ultrasound waves can provide enough energy to activate sensitizing agents and induce apoptosis, without the collateral damage to surrounding tissues seen with laser light in PDT. The aim of this initial study is to determine whether the sonosensitizers 5-ALA and IcG can be effectively delivered to, and detected in, the subcutaneous C6 glioma rat model.

## Methods

A total of 12 rats were inoculated with the C6 rat glioma cell line for use in this initial study. The rats were then split into three groups: 4 rats were treated with 100 mg/kg of intravenous 5-ALA, 4 rats were treated with 150 mg/mL of intravenous IcG, and 4 rats were used as a control. Rats given 5-ALA were euthanized 2 to 4 hours later. Tumors were then harvested for fluorescent spectroscopy study. In rats which were given IcG, we used *in vivo* imaging system to visualize the fluorescence intensity of the tumor as compared with the adjacent normal tissues.

## Results and conclusions

Tumor assays for all 8 of the experimental rats demonstrated uptake of the sensitizing agents. None of the assays for the control rats were positive. Our results confirm that 5-ALA and IcG were enriched in the subcutaneous C6 glioma in rats via the fluorescence spectroscopy or *in vivo* imaging. Future steps will include another proof-of-concept study to assess the ability of low-intensity ultrasound to cause hyperthermia-induced apoptosis in C6 gliomas via activation of 5-ALA and IcG. A larger study would then be needed compare the effectiveness of these sonosensitizers, and further optimize ultrasound parameters. Ultimately we would like to move this work towards a clinical trial.

